# Anxiety and depression after prostate cancer diagnosis and treatment: 5-year follow-up

**DOI:** 10.1038/sj.bjc.6603057

**Published:** 2006-04-04

**Authors:** I J Korfage, M-L Essink-Bot, A C J W Janssens, F H Schröder, H J de Koning

**Affiliations:** 1 Department of Public Health, Erasmus MC, University Medical Center Rotterdam, PO Box 1738, 3000 DR Rotterdam, The Netherlands; 2 Department of Urology, Erasmus MC, University Medical Center Rotterdam, PO Box 1738, 3000 DR Rotterdam, The Netherlands

**Keywords:** prostatic neoplasms, anxiety, depressive disorder, quality of life, screening, longitudinal studies

## Abstract

To document anxiety and depression from pretreatment till 5-year follow-up in 299 men with localized prostate cancer. To assess, if baseline scores were predictive for anxiety and depression at 1-year follow-up. Respondents completed four assessments (pretreatment, at 6 and 12 months, and at 5-year follow-up) on anxiety, depression and mental health. Respondents were subdivided according to therapy (prostatectomy or radiotherapy) and high *vs* low-anxiety. Pretreatment 28% of all patients were classified as ‘high-anxiety’; their average anxiety scores decreased significantly post-treatment, that is towards less anxiety. At all assessments, high-anxiety men treated by prostatectomy reported less depression than high-anxiety men treated by radiotherapy. Of men treated by radiotherapy, 27% reported clinical significant levels of depression while 20% is expected in a general population. The improvement in mental health at 6-months follow-up was statistically significant and clinically meaningful in all respondent groups. Sensitivity of anxiety at baseline as a screening tool was 71% for anxiety and 60% for symptoms of depression. We recommend clinicians to attempt early detection of patients at risk of high levels of anxiety and depression after prostate cancer diagnosis since prevalence is high. STAI-State can be a useful screening tool but needs further development.

Prostate cancer is highly prevalent in most Western countries (Parkin *et al*, 2001). Prostate cancer can be detected early by PSA-testing, a biologic tumour marker. Radical prostatectomy and external beam radiotherapy are the most commonly used intentionally curative primary therapies.

Being diagnosed with prostate cancer leads to anxiety, but not to the same extent in every patient (Balderson and Towell, 2003; Carlson *et al*, 2004; Steginga *et al*, 2004). In a recent study, some 30% of the participating prostate cancer patients met criteria for general distress in the clinical range (Carlson *et al*, 2004). In a retrospective, cross-sectional study among men with prostate cancer who were seeking psychological support, the prevalence of severe psychological distress was 37% (35/94) (Balderson and Towell, 2003). Although several longitudinal studies with a follow-up longer than 12 months have reported on mental health or emotional well-being after prostate cancer treatment (Potosky *et al*, 2000; Galbraith *et al*, 2001; Litwin *et al*, 2001, 2002; Korfage *et al*, 2005), the long-term impact of prostate cancer diagnosis and treatment on anxiety and feelings of depression in men is not known.

The high prevalence of severe distress after prostate cancer diagnosis has resulted in the recommendation to target interventions at treatment decision-related distress for all men and to offer in-depth psychological support for those who experience ongoing difficulties (Steginga *et al*, 2004). Yet, for clinicians and patients it would be useful if individual patients at risk of prolonged psychological distress could be identified shortly after diagnosis. To identify high-anxiety men who might need psychological support several instruments have been developed, for example the Memorial Anxiety Scale for Prostate Cancer (MAX-PC) (Roth *et al*, 2003) or the Hospital Anxiety and Depression Scale (HADS) (Nordin *et al*, 2001). Results were promising, but follow-up relatively short (2 weeks–6 months). We conducted a prospective study with 5-year follow-up in newly diagnosed prostate cancer patients to (1) document the course of anxiety and depression from before the initiation of treatment; and (2) evaluate the predictive accuracy of baseline scores at the individual level for anxiety and depression in the year following diagnosis.

## PATIENTS AND METHODS

### Ethics approval and informed consent

The ethics review committee of the Erasmus MC, the University Medical Center Rotterdam, the Netherlands, approved of the research protocol. All participating men gave written informed consent.

### Patients and procedures

All consecutive newly diagnosed prostate cancer patients from four Rotterdam hospitals were recruited between June 1996 and January 1999. Respondents were diagnosed through the ERSPC screening trial or in a clinical setting. Exclusion criteria were (1) referral to watchful waiting or advanced disease therapy; (2) noncompletion of the STAI-State scores (see below), since this score was crucial in assessing the respondents' anxiety level at baseline. The prospective study consisted of four measurements: 1 month before initiation of primary therapy, and at 6 months, 12 months and 5-year follow-up. The nonrandomly allocated treatment consisted of (intentionally nerve-sparing) radical prostatectomy or external beam radiotherapy (comprising an average of 33 radiation sessions over 7 weeks). Further details on the study design and the inclusion of respondents were published previously (Korfage *et al*, 2005).

### Patients' characteristics

Information on age, marital status, education, comorbidity and profession was obtained from the respondents. Educational level was classified as low (primary school or lower technical education), intermediate or high (college/university degree). To assess the prevalence of comorbidity, we used a standardized list of 28 chronic conditions, such as heart failure, asthma, diabetes and asked respondents to report which condition they currently experienced or had experienced during the previous year (Dutch Health Interview Survey, Statistics Netherlands).

Baseline clinical information on tumour stage (Hermanek and Sobin, 1992), histopathologic tumour (biopsy) grade and urologic treatment history were obtained from the Regional Cancer Registry. Possible postoperative adjustments to staging in the prostatectomy group were not included to maintain comparability with the radiotherapy group. Data on the clinical or biochemical progression at the 5-year assessment were obtained from the treating physicians. Biochemical recurrence was defined as a PSA-level of at least 0.2 ng ml^−1^ after prostatectomy, confirmed at least once, or as a rise in PSA-level of at least 0.5 ng ml^−1^ after radiotherapy, confirmed at least once.

### Psychological measures

The questionnaires contained three validated self-report psychological measures.

Anxiety was assessed by the State Trait Anxiety Inventory (STAI-State). This scale contains 20 items on, for instance, feeling at ease or upset (Sesti, 2000). Scale scores range from 20 to 80 with higher scores indicating higher levels of anxiety (van der Ploeg *et al*, 1980). A STAI-State score of more than 44 defines an individual as highly anxious (Spielberger and Vagg, 1984; Millar *et al*, 1995). Applying this cutoff level, we defined men with pretreatment STAI-State scores equal to or below 44 as ‘low-anxiety’ and those with scores above 44 as ‘high-anxiety’.

The Center for Epidemiologic Studies Depression Scale (CES-D) was used to assess the frequency and severity of symptoms of depression. We applied the 20-item version with items relating to, for instance, feeling depressed or fearful, being happy, and enjoying life. Scores range from 0 to 60 with higher scores indicating higher levels of symptoms of depression (Radloff, 1977). A score of 16 or higher suggests a clinically significant level of symptoms of depression, which does not necessarily mean that the participant has a clinical diagnosis of depression. In a general population sample, 20% of the participants had a CES-D score above 16 (Bouma *et al*, 1995).

The Mental Health scale of the RAND 36-item Short-Form Health Survey (SF36-MH) was used as a general measure of mental health. The scale consists of five items on being nervous, down, peaceful, depressed and happy. Item scores are transformed to ranges of 0–100 with higher scores indicating better mental health (Ware *et al*, 1993). Differences of at least 7.9 points are considered clinically meaningful (Norman *et al*, 2001).

Procedures concerning imputation of missing items were conducted according to the respective guidelines. STAI-State, CES-D and SF36-MH scores are moderately to strongly correlated ranging at baseline from 0.71 between STAI-State and CES-D to 0.83 between STAI-State and SF36-MH.

### Statistical analysis

Respondents were subdivided into four groups, defined by therapy (surgery or external radiotherapy) and by level of anxiety (high *vs* low). Between-group and within-group differences in background characteristics and descriptive statistics were calculated using SPSS for Windows, release 10.0.7. The *χ*^2^ test was used for categorical variables, the *t*-test or Mann–Whitney *U* for continuous variables. *P*-values <0.05 (referring to two-sided statistical tests) were considered significant.

The course of STAI-State, CES-D and SF36-MH scores within therapy groups was analysed by repeated-measures analysis of variance (ANOVA) using proc mixed from the SAS system for Windows release 8.2. Random intercept models were applied that allowed for the use of all available data, including incomplete records. These models comprised the main effects of ‘anxiety’ and ‘time’ and the interaction between anxiety and time. Time was included as a factor with four levels – one for each assessment - to account for possible nonlinearities in the course of scale scores. Comorbidity and PSA-level were included in the models as covariates.

To compare the course of anxiety and symptoms of depression in subgroups stratified by baseline anxiety scores, we calculated mean scores at 6 and 12-months follow-up for respondents with low (⩽44) and high (>44) anxiety scores at baseline. To evaluate the diagnostic performance of baseline anxiety screening, we calculated the sensitivity, specificity, positive (PPV) and negative predictive value (NPV) using data of all patients who completed the first three assessments. This complete case analysis is a valid strategy, because missing values at 1-year follow-up were not related to levels of anxiety or symptoms of depression at baseline nor to age.

### Age

Average age differed considerably between treatment groups (±6 years). Age-adjustment was not appropriate, because the relation between age and the physical functions in particular was found to be nonlinear (as the decline with ageing was generally steeper for older subjects than for younger ones). Therefore, the average age per treatment group is presented on the *x*-axes in our graphs – analogous to earlier reported disease-specific and generic quality of life scores of the same cohort (Korfage *et al*, 2005).

## RESULTS

### Patient's characteristics

Between June 1996 and January 1999, 415 men met the inclusion criteria, of whom 387 consented to participate and completed the first questionnaire (93%). Men who were referred to watchful waiting (*n*=25) or advanced disease therapy (*n*=48), or had not completed the STAI-State at the first assessment (*n*=15) were excluded. The final cohort consisted of 299 primary prostate cancer patients treated with radical prostatectomy (*n*=118) or external radiotherapy (*n*=181). The response rate to all four questionnaires was 78% (214 out of 275 men still alive at the 52-month assessment). Median time to long-term follow-up was 52 months, and mean time 51 months (range: 44–56 months).

Table 1 reports patients' characteristics per treatment group. The table shows that the groups differed significantly in age, the number of comorbid conditions, and the PSA-level before treatment. On the basis of the STAI-State scores at the first assessment, 25% of the prostatectomy (*n*=29) and 30% of the radiotherapy group (*n*=55) were classified as high-anxiety. Characteristics did not differ significantly between high-anxiety and low-anxiety individuals within the two treatment groups, except that in the radiotherapy group the percentage of singles was higher among high-anxiety men compared to low-anxiety men (26% *vs* 8%, *P*=0.003).

Information on clinical or biochemical recurrence at the 5-year assessment was available for 94% of the 5-year respondents; prostate cancer had recurred in 5% (5/91) of the prostatectomy patients and in 21% (26/123) of the radiotherapy patients.

### Psychological measures

Scores of men treated by surgery were more favourable than those treated by radiotherapy for all three psychological measures and at all four assessments, that is, less anxiety and feelings of depression and better mental health.

**Figure 1:**
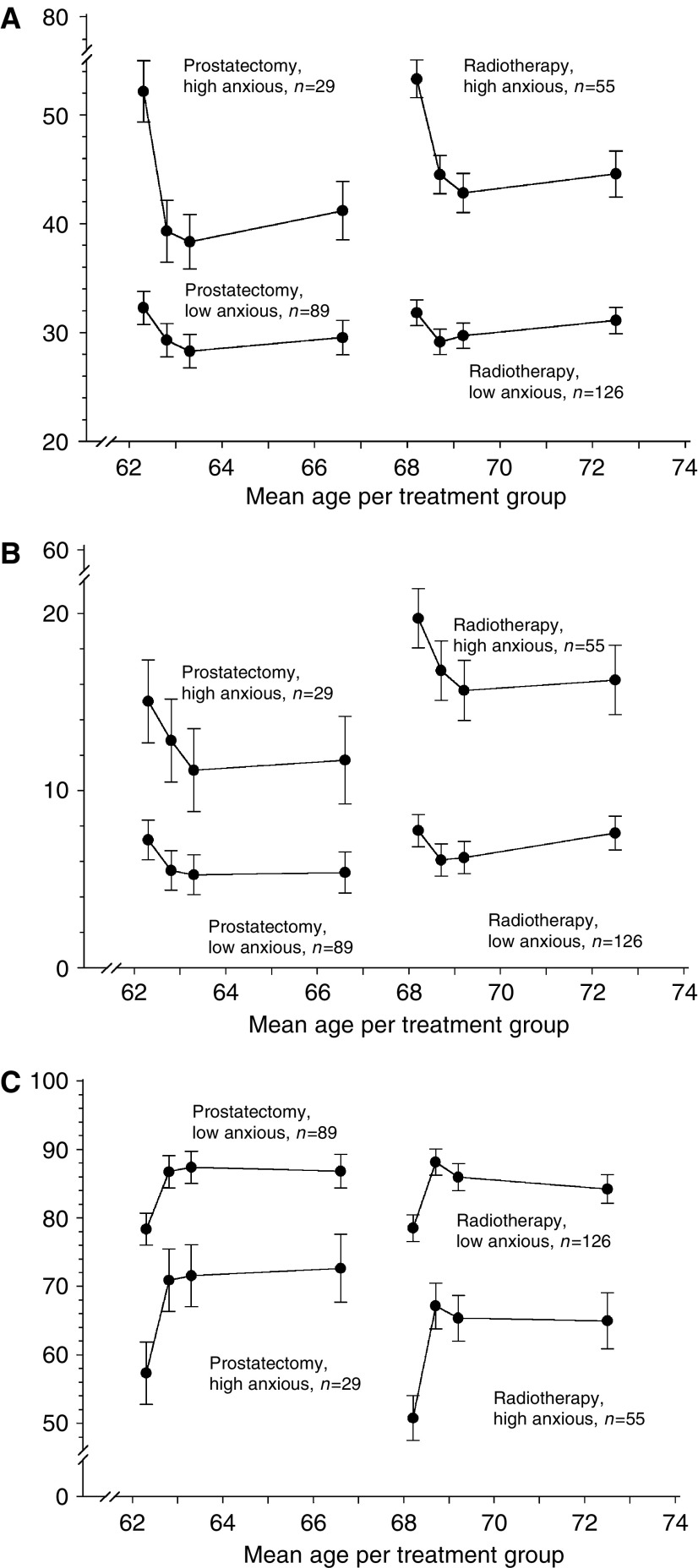
(**A**) STAI-State scores during 5-year follow-up by treatment group and baseline anxiety level. (**B**) CES-D scores during 5-year follow-up by treatment group and baseline anxiety level. (**C**) SF-36 Mental Health scores during 5-year follow-up by treatment group and baseline anxiety level.

Pretreatment, 28% of all patients reported clinical levels of anxiety (STAI-State >44), that is, 25% of the surgery group and 30% of the radiotherapy group. Average STAI-State scores in high-anxiety men of 52 (surgery group) and 54 (radiotherapy group) decreased significantly after treatment, that is, less anxiety, and remained at the lower level through follow-up (Table 2). At all assessments, surgically treated high-anxiety men reported less anxiety than high-anxiety men treated by radiotherapy. Repeated measures analysis showed a significantly different score pattern within treatment groups (*P*<0.0001): although high-anxiety men improved substantially at 6-months follow-up, they still reported more anxiety than low-anxiety men (Figure 1A).

Compared to pretreatment, all groups except the high-anxiety men treated by radiotherapy reported significantly lower CES-D scores at 6-months follow-up, that is, less symptoms of depression (Table 2). At all assessments, high-anxiety men treated by prostatectomy reported less feelings of depression than high-anxiety men after radiotherapy (Table 2). Repeated measures analysis showed that although levels of feelings of depression differed between high- and low-anxiety men, score patterns within treatment groups did not statistically differ (Figure 1B).

At all assessments *lower* percentages of prostatectomy men reported clinically significant levels of symptoms of depression than the general population (9–18% *vs* 20%), pretreatment and at 5-year assessments in the radiotherapy group these percentages were *higher* than 20% (27 and 22%).

Compared to pretreatment, the improvement in post-treatment SF36-MH was not only statistically significant, but also clinically meaningful (Ware *et al*, 1993) in all four respondent groups, see Table 2. High-anxiety prostatectomy men reported better mental health than high-anxiety radiotherapy men (Table 2). Repeated measures analysis showed that although levels of SF36-MH differed between high- and low-anxiety men, score patterns within treatment groups were not significantly different between the groups (Figure 1C).

### Predictive value of baseline scores

Table 3 shows the results of using the STAI-State score at baseline as a screening tool to predict high levels of anxiety and feelings of depression at the 6 and 12-months follow-up. The tool detected 71% of the patients who reported high anxiety at 6-months follow-up (sensitivity=71%), and 60% of those reporting a clinical level of symptoms of depression (sensitivity=60%). The probability of a high-anxiety score at 6 or 12-months follow-up was 42% in patients with a high-anxiety score at baseline (PPV=42%), and 8% in those with low-anxiety scores (NPV=92%). The probabilities of high levels of symptoms of depression at follow-up were 38% (PPV=38%) and 9% (NPV=91%) in these groups.

## DISCUSSION

We performed a prospective, longitudinal study on mental health in 299 prostate cancer patients, using validated instruments. At 1 month before treatment roughly one in every four patients was classified as ‘high-anxiety’. After 6 months, after initiation of treatment, men reported significantly less anxiety and feelings of depression and a significantly better mental health. Average scale scores remained at the improved levels through follow-up.

Before interpreting the results from a clinical perspective, two methodological issues have to be discussed. First, there is no unequivocal cutoff value for the STAI to define high-anxiety. We used the earlier reported cutoff value of 45 and higher (Millar *et al*, 1995; Roth *et al*, 1998) which matches almost perfectly with the validated cutoff value of the HADS (Millar *et al*, 1995). Second, nonresponse at 5-year follow-up was present. Although, repeated measures analyses can take incomplete cases into account, we excluded the 5-year assessment in evaluating the STAI-State as a screening tool; because the nonresponse at 5-year follow-up was significantly lower in high-anxiety men, a complete case analysis would not be appropriate. Furthermore, it can be argued that a prediction of high-anxiety at 12-months follow-up is clinically more relevant, because high levels of anxiety or symptoms of depression at 5-year follow-up may be unrelated to the preceding prostate cancer diagnosis and treatment.

When we selected an anxiety measure around 1996, validated Dutch versions of both STAI and HADS were available. We needed only one measure and chose STAI, but, in retrospect, we could have chosen HADS as well. We think that in this context there are no really strong scientific arguments to prefer the one to the other. Both are well-validated and commonly used measures for generic anxiety, each with their own strengths and weaknesses. The average STAI-State scores at baseline in the high-anxiety groups, that is, 52 for surgery patients and 54 for radiotherapy, are high in comparison to, for example, the mean STAI-State score in a group of males with anxiety neurosis of 45 (van der Ploeg *et al*, 1980). Of high-anxiety men, prostatectomy patients reported less anxiety and feelings of depression and better mental health at follow-up than radiotherapy patients. A first possible explanation is that the level of anxiety influenced the treatment decision; high-anxiety men may have perceived surgery as too frightening and therefore opted for radiotherapy. A second explanation could be that surgery led to more reassurance since – contrary to radiotherapy – the prostate actually is removed. Previous research has suggested that men may choose surgery on the basis of the lay belief that surgical removal is the most effective way to cure cancer (Steginga *et al*, 2002). A third explanation could be age, since high-anxiety radiotherapy men were significantly older than high-anxiety prostatectomy men. However, for a number of reasons, age does *not* seem to be the explanation. For instance, in spite of the difference in average age low-anxiety men who were treated by radiotherapy reported similar levels of anxiety and symptoms of depression as surgically treated low-anxiety men. Furthermore, several studies reported an association of higher age and lower levels of anxiety. A review study, for instance, reported some evidence that ageing is associated with an intrinsic reduction in susceptibility to anxiety and depression (Jorm, 2000). Furthermore, older men reported better mental health, although higher ages were associated with worse physical health (Litwin *et al*, 2002). And finally, higher levels of anxiety were found in prostate cancer patients younger than 65 years of age (Lintz *et al*, 2003). A fourth possible explanation could be that compared to the high-anxiety surgery group, a higher percentage of men were ‘single’ – that is, in most cases divorced or widowed – in the high-anxiety radiotherapy group. It has been reported before that marital status contributes to happiness (Joung *et al*, 1994). A fifth possible explanation could be the higher rate of recurrence in men treated by radiotherapy *vs* men treated by surgery, that is, 21 *vs* 5%. Within the radiotherapy group, the recurrence rate did not differ significantly between high- and low-anxiety men.

The frequency of side effects in this same group of patients has been reported elsewhere (Korfage *et al*, 2005). Four to five years post-treatment, side effects were reported by higher percentages of men treated by surgery than by men treated by radiotherapy, for example, 88% of erectile dysfunction *vs* 64%, and 31% of urinary leakage *vs* 13%. Thus side effects appear not to be the reason for higher anxiety levels in men treated by radiotherapy.

Our findings are in line with a cohort study on 111 prostate cancer patients with 12 months follow-up. Steginga *et al* (2004) found that psychological and treatment decision-related distress decreased with time, independent of treatment choice. At 12 months follow-up, most men experienced low levels of distress. The authors suggested that, in general, men are resilient to the experience of localized prostate cancer and adjust well psychologically. We agree that the majority of localized prostate cancer patients seem to do fine in the 1–5 years following treatment, but this is not the case for all patients. The challenge for clinicians is to detect those men early who will experience ongoing clinical levels of anxiety and symptoms of depression, and provide those with in-depth support. A (short) anxiety measure could be a useful tool. We applied a 20-item version of the STAI-State. Currently validated 6-item versions are available in English (Marteau *et al*, 2001) and other languages such as Dutch (van der Bij *et al*, 2003).

Vedana and co-workers compared the Hospital anxiety and depression scale (HADS) and STAI to identify the most suitable instrument for screening a population at in-hospital intensive rehabilitation on anxiety and depression. The sensitivity of the STAI (52%) was less than that of the HADS (72%), but its specificity (99%) was greater than that of HADS (84%). The authors concluded that both instruments can be recommended for psychological screening of patients in an in-hospital intensive rehabilitation (Vedana *et al*, 2002). A cross-sectional study on psychiatric disorders after successful renal transplantation assessed the value of self-report scales, among others STAI and HADS, in predicting anxiety and depression. HADS was found to significantly (*P*=0.003) predict anxiety and depression (Arapaslan *et al*, 2004).

In our study, using the STAI-State baseline score as a screening tool resulted in the early detection of 71, respectively, 60% of patients who were to experience high levels of symptoms of anxiety, respectively, feelings of depression at 6 or 12-months follow-up. The sensitivity might be improved by expanding the tool with, for instance, disease characteristics as the Gleason score, or by applying other measures, for instance the HADS (Zigmond and Snaith, 1983).

Treating clinicians may not always realize that, in spite of a comparably favourable prognosis, so many patients experience high levels of anxiety and symptoms of depression after a diagnosis of localized prostate cancer. We recommend clinicians to attempt early detection of patients at risk of such high levels and provide them with psychological support. STAI-State can be a useful screening tool but needs further development.

## Figures and Tables

**Table 1:** Patient characteristics at baseline

	**Prostatectomy (*n*=118)**	**Radiotherapy (*n*=181)**	***P*-value**
Age in years			<0.001
Average (s.d. range)	62.6 (5.3, 50–75)	68.1 (5.8, 50–82)	
			
*Educational level (%)*			0.10
Low	28% (31)	38% (66)	
Intermediate	57% (62)	54% (94)	
High	15% (16)	8% (14)	
			
*Marital status (%)*			1
Married or cohabiting	87% (103)	87% (155)	
Divorced or single	13% (15)	13% (24)	
			
*Comorbidity*			<0.001
Average number of conditions	0.7	1.2	
			
*PSA-level before treatment* (ng ml^−1^)			0.002
Average (s.d.)	9.7 (16.3)	15.7 (24.7)	
			
*Tumour stage before treatment*			0.06
T1	18% (19)	12% (20)	
T2	67% (71)	61% (103)	
T3	15% (16)	27% (45)	
T4	-	1% (2)	
			
*Tumour grade before treatment*			0.78
G1	51% (54)	50% (86)	
G2	38% (40)	37% (63)	
G3	11% (11)	13% (23)	

**Table 2:** Mean STAI-State, CES-D and SF36-MH by treatment and baseline anxiety level and standard deviation

	**Prostatectomy^a^**			**Radiotherapy^a^**		
	**High-anxiety (*n*=29)**	**Low-anxiety (*n*=89)**	**All (*n*=118)**	**High-anxiety (*n*=55)**	**Low-anxiety (*n*=126)**	**All (*n*=181)**
	**Mean (s.d.)**	**Mean (s.d.)**	**Mean (s.d.)**	**Mean (s.d.)**	**Mean (s.d.)**	**Mean (s.d.)**
STAI-State (80–20)						
Pretreatment	51.9 (7.8)	32.8 (7.5)	37.5 (11.2)	54.1 (7.7)	32.8 (6.5)	39.1 (11.7)
6 months	39.2^b^ (12.5)	29.8^b^ (8.4)	32.2^b^ (10.3)	45.6^b^ (11.8)	30.6^b^ (8.4)	34.9^b^ (11.5)
12 months	39.1 (11.6)	30.0 (8.7)	32.3 (10.2)	42.9 (11.4)	30.6 (9.7)	34.2 (11.6)
5 year	40.2 (11.3)	30.5 (8.4)	32.8 (10.1)	43.0 (10.3)	31.6^b^ (9.0)	34.4^b^ (10.5)
						
CES-D (60–0)						
Pretreatment	16.8 (9.0)	7.6 (5.9)	9.9 (7.8)	19.9 (8.7)	8.1 (5.8)	11.2 (7.9)
6 months	13.0^b^ (12.3)	5.9^b^ (7.1)	7.7^b^ (9.1)	17.3 (11.0)	6.5^b^ (6.1)	9.4^b^ (8.9)
12 months	11.6 (9.8)	5.9 (6.8)	7.4 (10.0)	15.0 (10.1)	6.5 (6.6)	8.9 (8.7)
5 year	10.8 (9.7)	6.1 (5.7)	7.3 (7.0)	15.5 (8.7)	7.5^b^ (7.2)	9.6^b^ (8.4)
						
SF36-MH (0-100)						
Pretreatment	55.4(13.3)	78.2 (11.5)	72.6 (15.5)	48.8 (14.3)	77.0 (11.2)	68.8 (17.2)
6 months	69.6^b^ (24.7)	86.0^b^ (13.9)	82.1^b^ (18.3)	64.7^b^ (22.3)	86.6^b^ (13.3)	80.3^b^ (18.9)
12 months	70.4 (21.3)	85.7 (14.2)	81.9 (17.4)	65.1 (20.8)	84.8 (15.4)	78.9 (19.4)
5 year	71.2 (19.5)	85.1 (12.9)	81.8 (15.7)	65.1 (21.9)	84.5 (15.7)	79.7 (19.2)

a Differences between anxious and nonanxious groups were <0.001 for all scale scores and at all assessments.

b Statistically different (*P*<0.05) from previous assessment.

**Table 3:** Evaluation of baseline anxiety screening for the prediction of psychological distress at follow-up

	**Anxiety score at baseline**					
	**Low (*n*=193)**	**High (*n*=69)**	**Sensitivity (%)**	**Specificity (%)**	**PPV (%)**	**NPV (%)**
*High-anxiety score*						
At 6 months	12 (6%)	29 (42%)	71	82	42	94
At 12 months	16 (8%)	26 (38%)	62	80	38	92
						
*High-depression score*						
At 6 months	17 (9%)	26 (38%)	60	80	38	91
At 12 months	18 (9%)	26 (38%)	60	80	38	91
						
*High-anxiety or -depression score*						
At 6 months	22 (11%)	34 (49%)	61	83	49	89
At 12 months	22 (11%)	33 (48%)	60	83	48	89
